# A Scoping Review of Exercises for Preventing Athletic Groin Pain

**DOI:** 10.7759/cureus.99883

**Published:** 2025-12-22

**Authors:** Hiromi Saito, Nadaka Hakariya, Tomoki Ebato, Norikazu Hirose

**Affiliations:** 1 Sports Medicine and Science, Waseda University, Tokyo, JPN; 2 Department of Life Sciences, Graduate School of Arts and Sciences, The University of Tokyo, Tokyo, JPN; 3 Department of Physical Education, International Pacific University, Okayama, JPN; 4 Faculty of Sport Sciences, Waseda University, Saitama, JPN

**Keywords:** chronic groin pain, copenhagen adduction exercise, groin injury, pubic bone, soccer injury

## Abstract

Groin pain is a frequent injury in multidirectional sports such as soccer, ice hockey, and Australian football. It commonly occurs during kicking, sprinting, and directional changes, yet preventive strategies remain poorly established. Although the Copenhagen Adduction Exercise (CAE) is widely adopted for groin pain prevention, evidence for other exercise options is limited. This scoping review aimed to identify and summarize exercise-based interventions used to prevent groin pain in athletes. The review followed the Preferred Reporting Items for Systematic reviews and Meta-Analyses extension for Scoping Reviews guidelines. Searches were conducted in PubMed, Web of Science, and PEDro (1983-April 2025) using the key concepts “groin pain,” “exercise,” and “prevention.” Only randomized and clinical trials published in English were included. After duplicate removal, 502 records were screened, and 19 studies met the eligibility criteria. Data were charted using Microsoft Excel, including study design, participant characteristics, intervention type, and outcomes. Two blinded reviewers assessed study quality using the PEDro scale. Descriptive and frequency-based analyses were performed to determine the most commonly implemented preventive exercises. In total, 19 randomized controlled trials were included, with an average PEDro score of 6.9 ± 1.2 (range = 5-9), indicating moderate methodological quality. The CAE was reported in 13 of 19 studies, showing improvements in adductor strength and, in some cases, reduced injury incidence. The Nordic hamstring exercise appeared in three studies, demonstrating benefits for strength and injury reduction. Additional programs such as the extended knee control program, abdominal and gluteus medius training, and trunk-hip coordination exercises were also identified. Counting the frequency of reported interventions revealed that CAE remains the most prevalent approach, while newer coordination-based or multi-joint programs show emerging evidence of benefit. Most studies focused on the CAE, indicating limited progress in groin pain prevention strategies. As CAE alone shows limited preventive effects, developing new, multifaceted exercise approaches is essential. This review was retrospectively registered in the Open Science Framework, relied solely on English-language publications, and included only published trials, which may introduce selection and publication bias.

## Introduction and background

Groin pain is a common injury in soccer [1]. It has been reported that 59% of male soccer players have experienced groin pain in the past [1]. This injury often occurs during kicking, changing direction, and sprinting [2], and can occur in various sports. It is a common condition in multidirectional sports and is often characterized by a recurrent or persistent clinical course, which can limit performance and lead to prolonged time loss from sport. Given these reports, preventing groin pain is extremely important.

Exercise interventions are generally the first choice for preventing groin pain. A systematic review on exercises for groin pain prevention indicates limited evidence that exercise interventions reduce the incidence and risk of groin injuries in athletes [3], suggesting exercise remains an unexplored area in groin pain prevention. Furthermore, among exercise-based strategies for groin pain prevention, the Copenhagen Adductor Exercise (CAE) has been most widely implemented. The CAE is a partner-assisted eccentric strengthening exercise that primarily targets the hip adductor muscles and has been incorporated into preventive training programs across various sports [4]. Based on this evidence, the CAE is the only widely adopted exercise for groin pain prevention, and its effectiveness is not high among the many available exercise protocols. Therefore, it is important to identify exercises recognized as effective for groin pain prevention and provide clinicians with a broader range of options.

In contrast to groin pain prevention, hamstring injury prevention is supported by a wide range of well-established exercise options. Because the hamstrings are biarticular muscles spanning both the hip and knee joints, preventive programs include joint-specific exercises such as hip-dominant movements (e.g., deadlifts) and knee-dominant exercises (e.g., Nordic hamstring (NH) exercises) [5]. Consequently, preventive strategies for hamstring injuries are more diverse and clearly structured than those currently available for groin pain.

Therefore, the objective of this scoping review was to map and categorize exercise-based interventions for the prevention of sports-related groin pain in athletes, with a particular focus on identifying the most commonly implemented exercises across published studies.

## Review

Methodology

**Figure 1:**
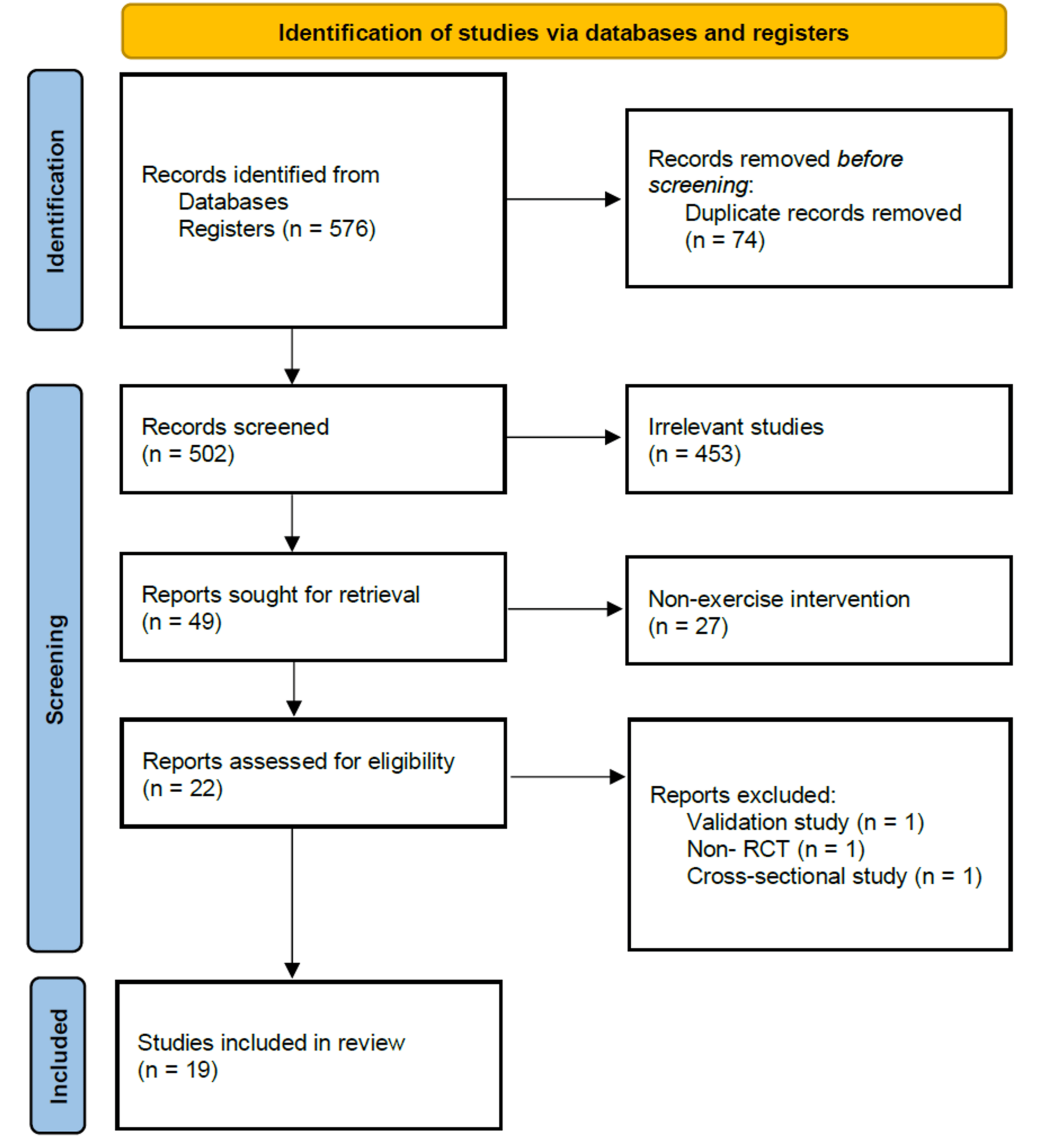
Preferred Reporting Items for Systematic Reviews and Meta-Analysis extension for Scoping Reviews flow diagram.

This scoping review was conducted in accordance with the Preferred Reporting Items for Systematic reviews and Meta-Analyses extension for Scoping Reviews (PRISMA-ScR) (Figure 1). The research question of this study focused on identifying the types of exercise intervention programs available for the prevention of groin pain during exercise and their effectiveness. The literature search was conducted using PubMed (via MEDLINE), Web of Science Core Collection, and the Physiotherapy Evidence Database (PEDro) covering the period from 1983 to 2025.

The search strategy used the following three categories: (1) groin pain, (2) exercise, and (3) prevention, combined using Boolean operators (the search strategy is detailed in the Appendices). Eligibility criteria included clinical trials published in English by April 15, 2025. All age groups were included. Studies were selected based on the titles and abstracts of the identified papers, and two independent reviewers assessed their relevance and eligibility. First, a total of 576 data items were collected. There were 394 items in PubMed, 168 items in Web of Science, and 14 items in PEDro. After entering the search terms, all data were saved in RefWorks. Subsequently, duplicate papers were deleted using RefWorks’ automatic duplicate removal system. There were 74 duplicate papers, leaving 502 studies for screening. Inter-rater agreement for study inclusion was substantial (κ = 0.953). In cases where the two reviewers did not agree, a discussion was held, and, ultimately, a consensus was reached on all included studies. Data were extracted from the selected studies using a standardized data extraction form. Information such as the title, authors, and publication year was collected from the articles. This review was registered retrospectively in the Open Science Framework (OSF) according to the PRISMA-SCR guidelines. The protocol is available at https://osf.io/2mqsx/overview. No prospective protocol was registered before conducting the review. Hand searches of relevant publications were not performed to maintain consistency. Contact was not made with the study authors regarding unpublished data. The most recent database search was conducted on April 15, 2025.

Eligibility Criteria, Paper Review, and Exclusion Criteria

This scoping review followed the Population-Concept-Context (PCC) framework in accordance with the PRISMA-ScR guidelines. The PCC framework was defined as follows: the population comprised healthy athletes; the concept focused on exercise-based interventions used for the prevention of groin pain, with particular emphasis on identifying which exercises are most commonly implemented; and the context included sports and athletic training settings. Regarding the characteristics of the sources of evidence, eligible studies were peer-reviewed journal articles published between 1983 and April 15, 2025. The starting year (1983) corresponds to the earliest publication identified through the database searches that met the eligibility criteria. Only studies published in the English language were included. This language restriction was applied to ensure accurate data extraction and consistent interpretation of methodological details. The potential exclusion of relevant studies published in other languages is acknowledged as a limitation of this review. With respect to publication status, only full-text articles published in peer-reviewed journals were considered eligible. Conference abstracts, dissertations, non-peer-reviewed reports, and other forms of gray literature were excluded to maintain methodological consistency and transparency. One researcher independently reviewed the titles and abstracts obtained from the initial literature search based on their relevance to the purpose of the scoping review. Review articles and meta-analyses were excluded, and only randomized controlled trials and clinical trials were included. Full-text articles were then carefully reviewed, and studies targeting athletes and those involving exercise interventions for the prevention of groin pain were selected. Excluded papers included studies targeting non-athletes, studies involving surgical interventions, studies using braces or taping, case reports, and non-randomized controlled trials.

Data Collection

Data charting was performed using a standardized form created in Microsoft Excel. The form was designed to record key study information, including author, publication year, participant characteristics, type of sport, intervention details (author, year, age, group exercise outcome), and main outcomes related to groin pain prevention. The charting form was not pilot-tested before use. All data were extracted by a single reviewer. Because the extracted information was sufficiently detailed in the original articles, no contact with study authors was necessary to obtain or confirm additional data.

Critical Appraisal of Individual Sources of Evidence

The PEDro scale is an 11-item tool for assessing the quality of evidence [6]. Scores below 4 are considered “low quality,” 5-6 are “moderate,” and 7 or above are “high quality” [6]. Using the PEDro scale allows for the reliable and valid assessment of the methodological quality of randomized controlled trials [6]. Two blinded reviewers evaluated each piece of valid evidence. The PEDro scale was used to evaluate the evidence. It is one of the scales widely used in the field of physical therapy to evaluate randomized controlled trials. When there was disagreement on the evaluation items, the two reviewers discussed and made a final decision. Methodological quality was assessed using the PEDro scale by two independent reviewers. Before the assessment, both reviewers were familiarized with the PEDro scale and its scoring criteria using the official PEDro guidelines. Any disagreements in PEDro item scores were resolved through discussion, and consensus was reached for all items included in the final quality assessment.

Data Handling and Summarizing

Extracted data were organized in Microsoft Excel and summarized descriptively. The included studies were categorized according to intervention type (e.g., adductor strengthening, coordination-based exercises), participant characteristics, and sport type.

In addition to narrative synthesis, the frequency of each type of preventive exercise reported across studies was counted to identify which exercises were most commonly implemented. This frequency-based qualitative comparison allowed identification of exercises that are widely applied for groin pain prevention among athletes.

Quantitative data such as participant numbers and intervention duration were summarized using simple descriptive statistics, and heterogeneity among studies in terms of population and intervention design was considered qualitatively during interpretation of the results.

Results

Of the 502 potentially relevant papers identified through the search strategy, 453 were excluded after the first-level screening of titles and abstracts. This first-level screening excluded review studies, systematic review studies, studies of surgical interventions, intervention studies using taping or bracing, and case reports. The second-level screening excluded studies in which the participants were patients with groin pain, studies on the reliability and validity of exercise interventions, and non-randomized studies. As a result of the screening, 19 studies on the prevention of groin pain using randomized controlled trials were identified [7-25]. Of the papers reviewed, nine reported outcomes related to muscle strength [9-11,14,15,17,20,22,23,25], two reported the Oslo Trauma Injury Scale [12,24], five reported injury incidence and types [7,8,13,18,21], two reported muscle thickness and flexibility using ultrasound [16,19], and one study reported on lumbo-pelvic stability [15] (Table 1).

**Table 1:** A summary of included studies. HR: high-risk; LR: low risk; NH: Nordic hamstring exercise; HSR: heavy strength training; EHAD: maximal eccentric hip-adduction strength; IHAD: maximal isometric hip-adduction strength; IHAB: maximal isometric hip-abduction; CAE: Copenhagen Adduction Exercise; LABM: maximal endurance of the lateral abdominal musculature; EHAB: maximal eccentric hip abduction strength; OSTRC: Oslo Sports Trauma Research Centre Overuse Injury Questionnaire; HAGOS: Copenhagen Hip and Groin Outcome Score; FADIR: flexion, adduction, internal rotation test; BKFO: bent knee fall-out test; GPS: groin pain syndrome; IPEP: injury prevention exercise program; LVG: warm-up low-volume; HVG: warm-up high-volume; SQ: adductor squeeze

Author	Age	Group	Exercise	Outcome
Engebretsen et al., 2008 [7]	N/A	HR intervention (n = 193)	Ankle, knee, and groin training programs (adductor squeeze, NH)	Time loss injury, Severity of injury
		HR control (n = 195)		
		LR control (n = 120)		
Hölmich et al., 2010 [8]	Prevention group: 24.5	Prevention group (n = 477)	Concurrent contraction of the adductor muscles and the abdominal muscles	Time until the first groin injury
	Control group: 24.6	Control group (n = 430)		
Jensen et al., 2014 [9]	N/A	HSR group (n = 13)	Hip-adductor training with elastic bands	Maximal eccentric hip-adduction strength, maximal isometric hip-adduction strength, maximal isometric hip-abduction
		Control group (n = 11)		
Ishøi et al., 2016 [10]	Intervention: 17.3 years (17–18 years)	Intervention (n = 10)	CAE	LABM, EHAB
	Control group: 17.4 years (17–18 years)	Control group (n = 10)		
Haroy et al., 2017 [11]	NH Group: 16.9 (1.0)	NH group (n = 16)	Including the NH in the FIFA 11+, Including the CAE in the FIFA 11+	Eccentric hip adduction strength test
	CAE Group: 16.7 (0.9)	CAE group (n = 17)		
Haroy et al., 2019 [12]	Intervention: 22.0 ± 4.3	Intervention group (n = 247)	CAE	OSTRC
	Control: 23.7 ± 4.3	Control group (n = 242)		
Beaudouin et al., 2019 [13]	Intervention group: 11.7 ± 0.8	Intervention group (n = 2,066)	11+ kids’ injury prevention program	Number of injuries, Severity of injuries
	Control group: 11.3 ± 1.2	Control group (n = 1,829)		
Kohavi et al., 2020 [14]	N/A	CAE (n = 14)	CAE, sliding hip	EHAD/EHAB
		Sliding hip (n = 16)		
		Control group (n = 12)		
Guerrero et al., 2021 [15]	Experimental group: 23.9 ± 2.8	Experimental group (n = 13)	Abdominal and gluteus medius training	Lumbo-pelvic stability, adductor muscle strength
	Control group: 25.8 ± 3.2	Control group (n = 12)		
Alonso et al., 2021 [16]	N/A	Intervention group (n = 6)	CAE	Ultrasound imaging of the adductor muscle
		Control group (n = 6)		
Dawkins et al., 2021 [17]	Intervention group: 19.5 ± 1.2	Intervention group (n = 20)	Low-intensity CAE	Eccentric adduction strength, adductor squeeze strength
	Control group: 19.3 ± 1.0	Control group (n = 19)		
Fujisaki et al., 2022 [18]	CAE:16.4 ± 0.9	CAE (n = 66)	CAE, NH	Injury report
	CAE and NH: 16.0 ± 0.7	CAE and NH (n = 73)		
	Control group:16.1 ± 0.9	Control group (n = 63)		
Alonso et al., 2022 [19]	Experimental group: 26.3 ± 2.9	Experimental group (n = 25)	CAE	Ultrasound imaging of the adductor muscle
	Control group: 25.8 ± 3.1	Control group (n = 20)		
Cotellessa et al., 2023 [20]	Treatment group: 16.8 ± 0.5	Treatment group (n = 21)	CAE	HAGOS, squeeze test, FADIR, BKFO, hip adduction strength
	Control Group: 17.1±0.8	Control Group (n=21)		
Lindblom et al., 2023 [21]	Extended knee control: 19.3 ± 5.0	Extended knee control (n = 197)	Extended knee control, adductor programme (including CAE)	Weekly questionnaires about football exposures and injuries
	Adductor programme: 20.5 ± 5.8	Adductor programme (n = 125)		
	Comparison group 20.5±6.3	Control group (n=180)		
Quintana-Cepedal et al., 2024 [22]	LVG: 14 ± 0	LVG (n = 13)	CAE	The five-second squeeze test
	HVG: 14 ± 0	HVG (n = 8)		
	Controls: 14.5 ± 0.52	Control group (n = 9)		
Pippas et al., 2024 [23]	CAE group: 16.7 ± 0.9	CAE group (n = 30)	CAE, adductor squeeze exercise	Maximal EHAD
	SQ group: 16.4 ± 0.8	SQ group (n = 27)		
Gram et al., 2025 [24]	Intervention group:14.0 ± 2.0	Intervention group (n = 119)	Rhythmic gymnastics-specific, injury prevention program	OSTRC
	Control group: 13.6 ± 2.0	Control group (n = 84)		
Thorarinsdottir et al., 2025 [25]	Low-volume group: 19.4 ± 3.1	Low-volume group (n = 22)	CAE	Maximal Isometric hip adductor strength testing
	High-volume group: 19.0 ± 1.8	High-volume group (n = 18)		

Quality of Evidence

The mean PEDro score was 6.89 ± 1.24. The minimum score was 5, and the maximum score was 9. The average score indicated that the quality of the collected literature was average (Table 2).

**Table 2:** Physiotherapy Evidence Database (PEDro) scores.

Study	1. Eligibility criteria	2. Randomized group allocation	3. Allocation Concealment	4. Similarity of Prognosis Prediction Measurements	5. Blinding of all participants	6. Therapist blinding	7. Blinding of evaluators	8. Data collected from over 85% of participants	9. Data collection from all participants or “intention to treat” analysis	10. Results of intergroup statistical comparisons	11. Provides both point measurements and variability measurements	Total
Engebretsen et al., 2008 [7]	Yes	Yes	No	No	No	No	No	Yes	No	Yes	Yes	5
Hölmich et al., 2010 [8]	NO	Yes	Yes	No	No	No	Yes	Yes	No	No	Yes	5
Jensen et al., 2014 [9]	Yes	Yes	Yes	Yes	No	No	No	No	No	Yes	Yes	6
Ishøi et al., 2016 [10]	Yes	Yes	Yes	No	No	No	Yes	No	No	Yes	Yes	6
Haroy et al., 2017 [11]	Yes	Yes	Yes	Yes	No	No	Yes	No	No	Yes	Yes	7
Haroy et al., 2019 [12]	Yes	Yes	Yes	Yes	No	No	Yes	No	Yes	Yes	Yes	8
Beaudouin et al., 2019 [13]	Yes	Yes	Yes	No	No	No	Yes	Yes	No	Yes	Yes	7
Kohavi et al., 2020 [14]	Yes	Yes	Yes	Yes	No	No	Yes	No	No	Yes	Yes	7
Guerrero et al., 2021 [15]	Yes	Yes	No	Yes	No	No	Yes	Yes	Yes	Yes	Yes	8
Alonso et al., 2021 [16]	Yes	Yes	No	Yes	No	No	No	No	No	Yes	Yes	5
Dawkins et al., 2021 [17]	Yes	Yes	Yes	Yes	No	No	Yes	Yes	Yes	Yes	Yes	9
Fujisaki et al., 2022 [18]	NO	Yes	Yes	No	No	No	No	Yes	Yes	Yes	Yes	6
Alonso et al., 2022 [19]	Yes	Yes	No	Yes	No	No	Yes	Yes	Yes	Yes	Yes	8
Cotellessa et al., 2023 [20]	Yes	Yes	Yes	No	No	No	Yes	Yes	Yes	Yes	Yes	8
Lindblom et al., 2023 [21]	Yes	Yes	No	Yes	No	No	Yes	No	Yes	Yes	Yes	7
Quintana-Cepedal et al., 2024 [22]	Yes	Yes	Yes	Yes	Yes	No	No	No	No	Yes	Yes	7
Pippas et al., 2024 [23]	Yes	Yes	Yes	Yes	No	No	Yes	No	No	Yes	Yes	7
Gram et al., 2025 [24]	Yes	Yes	Yes	Yes	No	No	Yes	Yes	Yes	Yes	Yes	9
Thorarinsdottir et al., 2025 [25]	Yes	Yes	Yes	No	No	No	Yes	No	No	Yes	Yes	6

Copenhagen Abduction Exercise

CAE was used in 13 out of 19 studies [10,11,12,14,16-23,25]. Among studies that included CAE as an exercise intervention, eight showed measuring muscle strength [10-12,14,17,20,22,23,25]. Two studies reported improvements in pain tests and questionnaires [12,21]. One study reported a reduction in the number of injuries [18], and two studies reported improvements in muscle thickness and flexibility [16,19].

Nordic Hamstring Exercise

Three out of 19 studies included NH in their interventions [7,11,18]. The breakdown is as follows: one study reported improvements in muscle strength [11], and two studies reported a reduction in injury incidence [7,18].

Other

Other interventions included the extended knee control program [21], abdominal and gluteus medius training [15], rhythmic gymnastics-specific injury prevention program [24], and trunk and hip exercises [8]. The extended knee control program reported a reduction in the number of injuries and reported that it was as effective as CAE in preventing injuries [21]. Abdominal and gluteus medius training reported improvements in trunk and gluteal muscle strength, and trunk and hip exercises reported a reduction in the incidence of groin pain [15,21].

Discussion

This scoping review comprehensively mapped randomized controlled trials evaluating exercise-based interventions for the prevention of groin pain in athletes. The findings indicate that the CAE remains the most commonly used approach, while a growing number of studies have explored the effectiveness of NH and control-based programs. To our knowledge, this is the first review to systematically compare these interventions across multiple outcome categories, including strength, injury incidence, and muscle morphology.

In recent years, there have been many reports on CAE and NH for the prevention of groin pain. Most exercises reported so far are mainly CAE, indicating that further development of exercises for the prevention of groin pain is important. For hamstring strains, exercises such as NH exercises, single-leg Romanian dead lifts, and prone hip extensions are used for injury prevention [5]. Furthermore, exercises to prevent ankle sprains are mostly prescribed as a combination of hopping exercises [26] and lateral hip strengthening exercises [26]. Given the evidence from studies on other sports injuries, further in-depth investigation into the factors that prevent groin pain may be warranted.

The effectiveness of CAE seems to be supported by the strengthening of the adductor muscles [10]. The preventive effects of CAE may be partially attributed to improved co-activation with the oblique muscles, given their anatomical and functional linkage to the adductors. However, this hypothesis remains speculative and requires further electromyographic and kinematic investigation to clarify the underlying mechanisms. The muscle functions of the adductor muscles and external oblique muscles are very important in kicking and changing direction [2], which are movements that cause groin pain [2]. It is possible that improving the function of this muscle connection improves the onset of groin pain and increases the stability of the support leg, resulting in improved adductor muscle strength. However, recent studies indicate that while CAE strengthens the adductor muscles, it does not prevent the occurrence of injuries themselves [3]. This suggests that although CAE is widely adopted for groin pain prevention, its effectiveness is actually limited, highlighting the urgent need to develop new preventive exercises.

The multifunctionality of the adductor longus muscle may enhance muscle strength through the NH intervention and reduce the incidence of groin pain. The adductor also plays a role in hip flexion and extension [18]. The adductor muscles and hamstrings assist in hip adduction, and, conversely, the adductor muscles assist in hip extension [9]. These findings suggest that combining CAE and NH may provide complementary benefits by addressing both frontal and sagittal plane muscle demands, particularly relevant in sports involving sprinting and directional changes. This integrated approach may therefore offer a more comprehensive prevention strategy. Kicking and sprinting, which are movements that cause groin pain, are mainly sagittal plane movements, and strengthening the hamstrings may reduce the load on the adductor muscles.

The extended knee control program has been reported to be as effective as CAE in preventing groin pain [12]. Abdominal and gluteus medius training has been shown to improve adductor muscle strength and lumbar spine and pelvic stability [21]. A decrease in the incidence of groin pain has been reported with trunk and hip exercises. The extended knee control program may offer preventive benefits by enhancing neuromuscular control and optimizing trunk-hip coordination, thereby reducing compensatory stress on the adductor region. Its emphasis on dynamic movement patterns suggests that improving movement quality, rather than solely increasing muscle strength, could play a key role in the prevention of groin pain. Previous research reported that improving error movements was effective in the treatment of groin pain, rather than focusing on improving muscle strength [27]. Thus, exercises based on the context of movements that cause groin pain may be effective in preventing groin pain.

Limitations and future research

In this study, it was not possible to standardize the results because we identified exercises used to prevent groin pain. Therefore, it was difficult to clearly present the results using systematic review and meta-analysis methods. To directly report the preventive effect of groin pain, it is necessary to report the number of occurrences and injury rates. Therefore, the findings should be interpreted as a descriptive overview of existing preventive exercise strategies rather than evidence of efficacy. Furthermore, the studies included in this review included athletes of different skill levels, sports, ages, and genders. The frequency and prevalence of groin pain vary by competitive level, incidence rates differ by sport, and onset is influenced by age and gender. A limitation of this study is that these demographic characteristics could not be standardized. This study conducted a scoping review based on English-language sources only and therefore could not evaluate articles written in other languages. Furthermore, only published articles were included, and therefore, publication bias is likely to be present. This review was retrospectively registered in OSF. The lack of prospective registration represents a methodological limitation; however, the review process followed PRISMA-SCR standards, and all methods have been documented to ensure transparency. Although duplicate data extraction was not applied to all studies from the initial phase, this limitation was mitigated by independent double extraction in later stages and consensus-based resolution of discrepancies.

## Conclusions

The majority of studies included in this review focused on CAE, suggesting that preventive exercise strategies for groin pain have seen little progress since the introduction of CAE. The combination of CAE and NH may play a significant role in preventing future groin injuries. Specifically, acquiring intermuscular coordination could enhance support function during movement and prove effective in injury prevention. Furthermore, recent years have seen the emergence of new exercises for groin pain prevention, such as the extended knee control protocol. This suggests that focusing on improving movement quality and reducing load on the groin from other joints may be important going forward. Given the currently limited preventive effect of CAE alone, the development of new, multifaceted exercise approaches is urgently needed.

## Appendices

**Table 3:** Search query used for searching.

Pub med
((groin pain) OR (inguinal pain) OR (adductor pain)) AND ((exercise) OR ((training) OR (physical therapy)) OR (rehabilitation)) AND ((prevention) OR (prophylaxis) OR (preventive measures))
Web of Science
((groin pain) OR (inguinal pain) OR (adductor pain)) AND ((exercise) OR (training) OR( physical therapy OR rehabilitation)) AND ((prevention) OR (prophylaxis) OR (preventive measures))
Database: PEDro
Search strategy: Advanced
Abstract and Title: groin
Body part: thigh or hip
Subdiscipline: Sports
Method: clinical trial
